# Supplementary figures and images for: Host plants selection of Centranthera grandiflora Benth. and nontargeted metabolomics analysis of its parasitic and non-parasitic samples
Source: PLoS One. 2025 Feb 5;20(2):e0310786. doi: 10.1371/journal.pone.0310786 (PMC11798490; doi:10.1371/journal.pone.0310786)

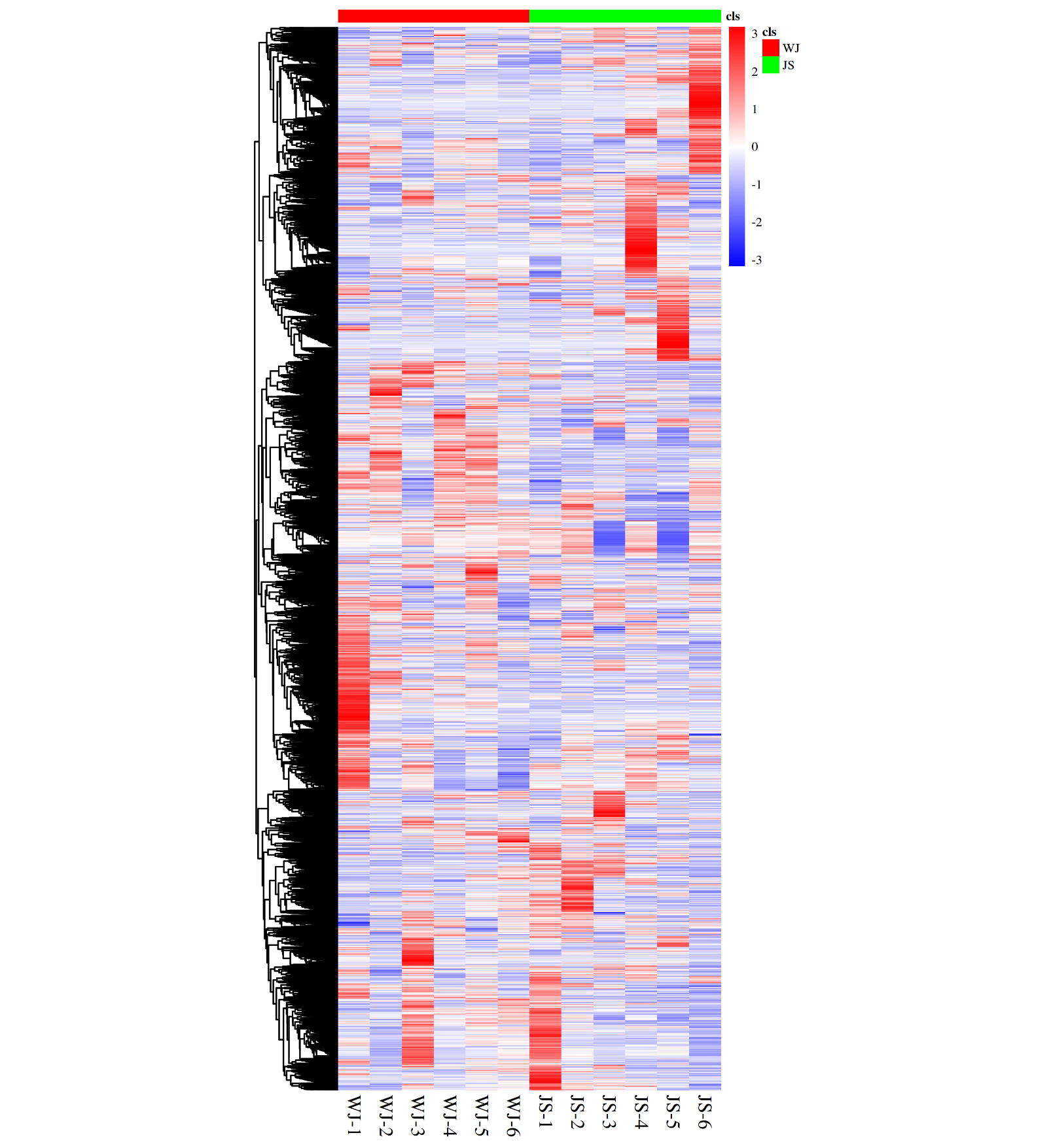


Figure S1 Heatmap of overall metabolites in positive ion mode

Supplement: S1 Fig — (DOCX) [file pone.0310786.s001.docx]

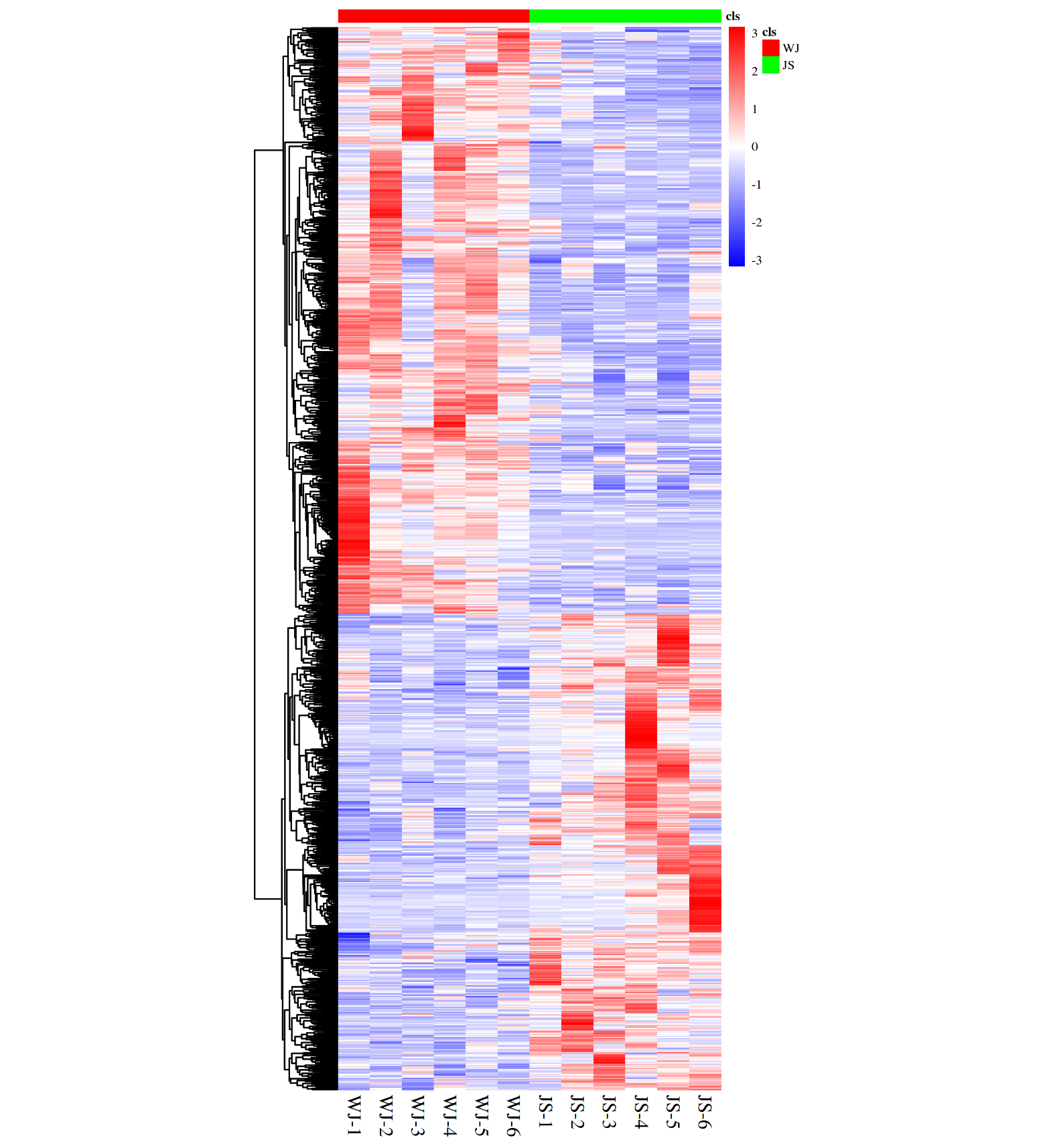


Figure S2 Heatmap of differential metabolites of WJ and JS samples in positive ion mode

Supplement: S2 Fig — (DOCX) [file pone.0310786.s002.docx]

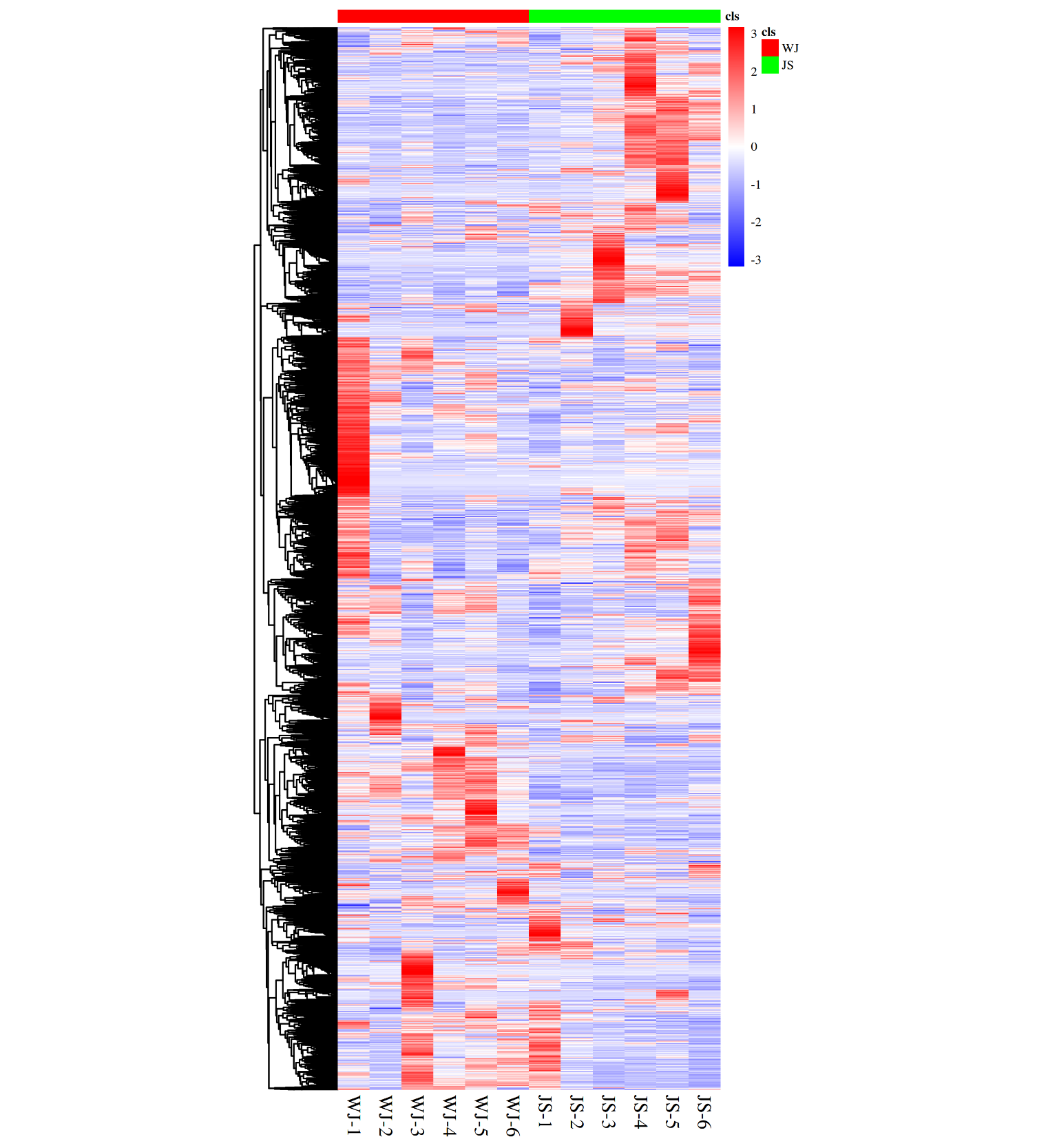


Figure S3 Heatmap of overall metabolites in negative ion mode

Supplement: S3 Fig — (DOCX) [file pone.0310786.s003.docx]

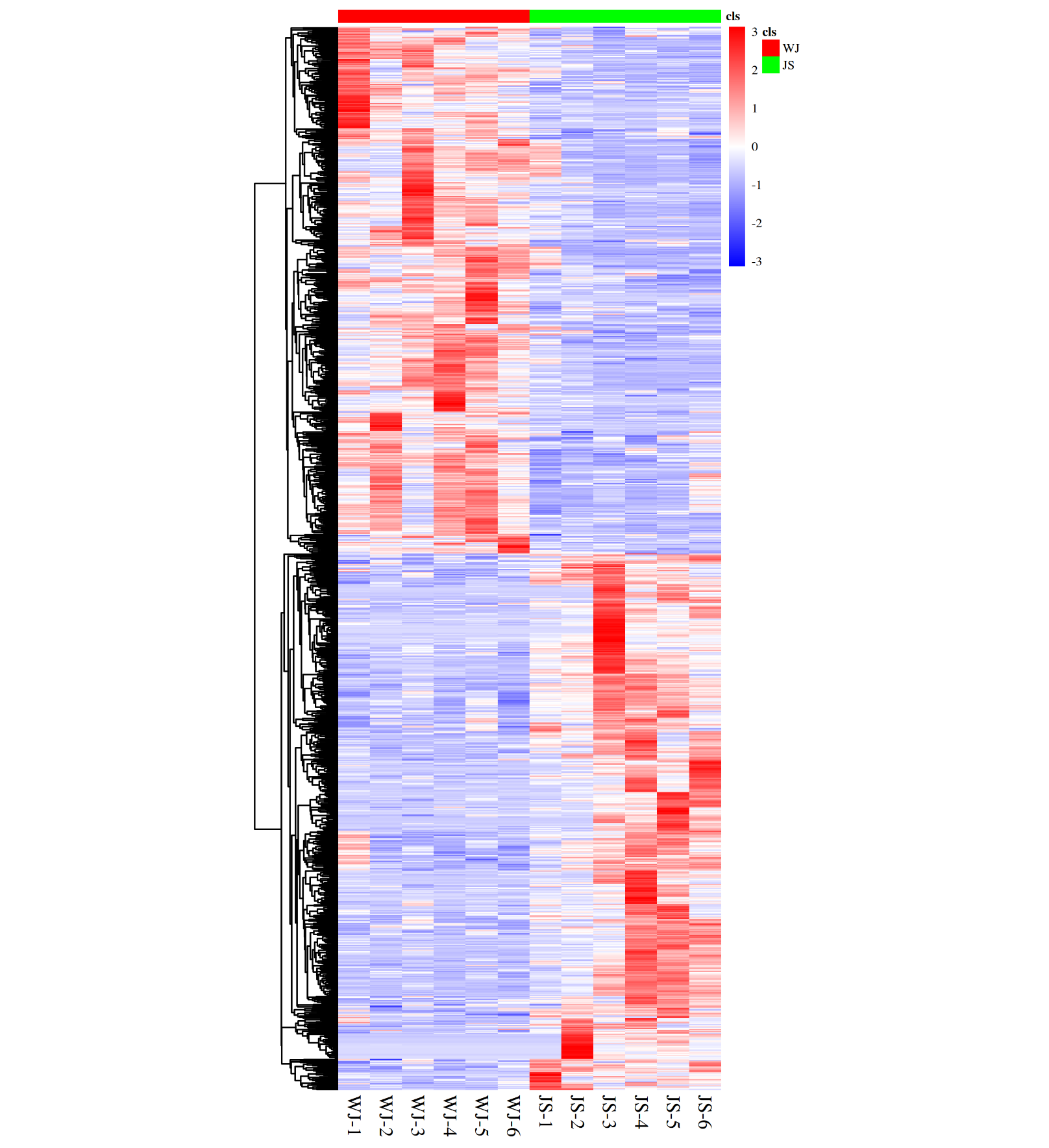


Figure S4 Heatmap of differential metabolites of WJ and JS samples in positive ion mode

Supplement: S4 Fig — (DOCX) [file pone.0310786.s004.docx]

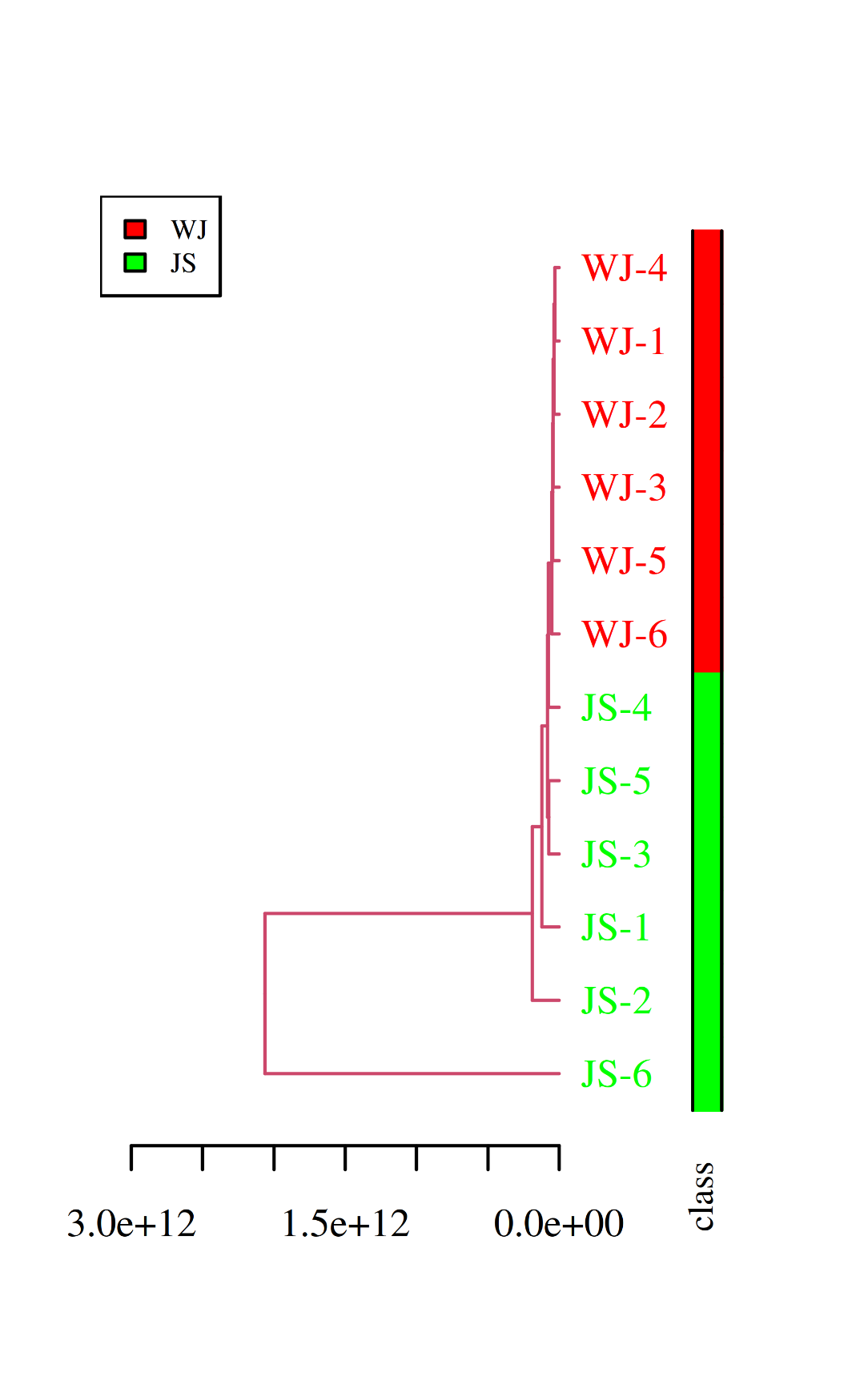


Figure S5 Dendrogram of the overall samples in positive ion mode

Supplement: S5 Fig — (DOCX) [file pone.0310786.s005.docx]

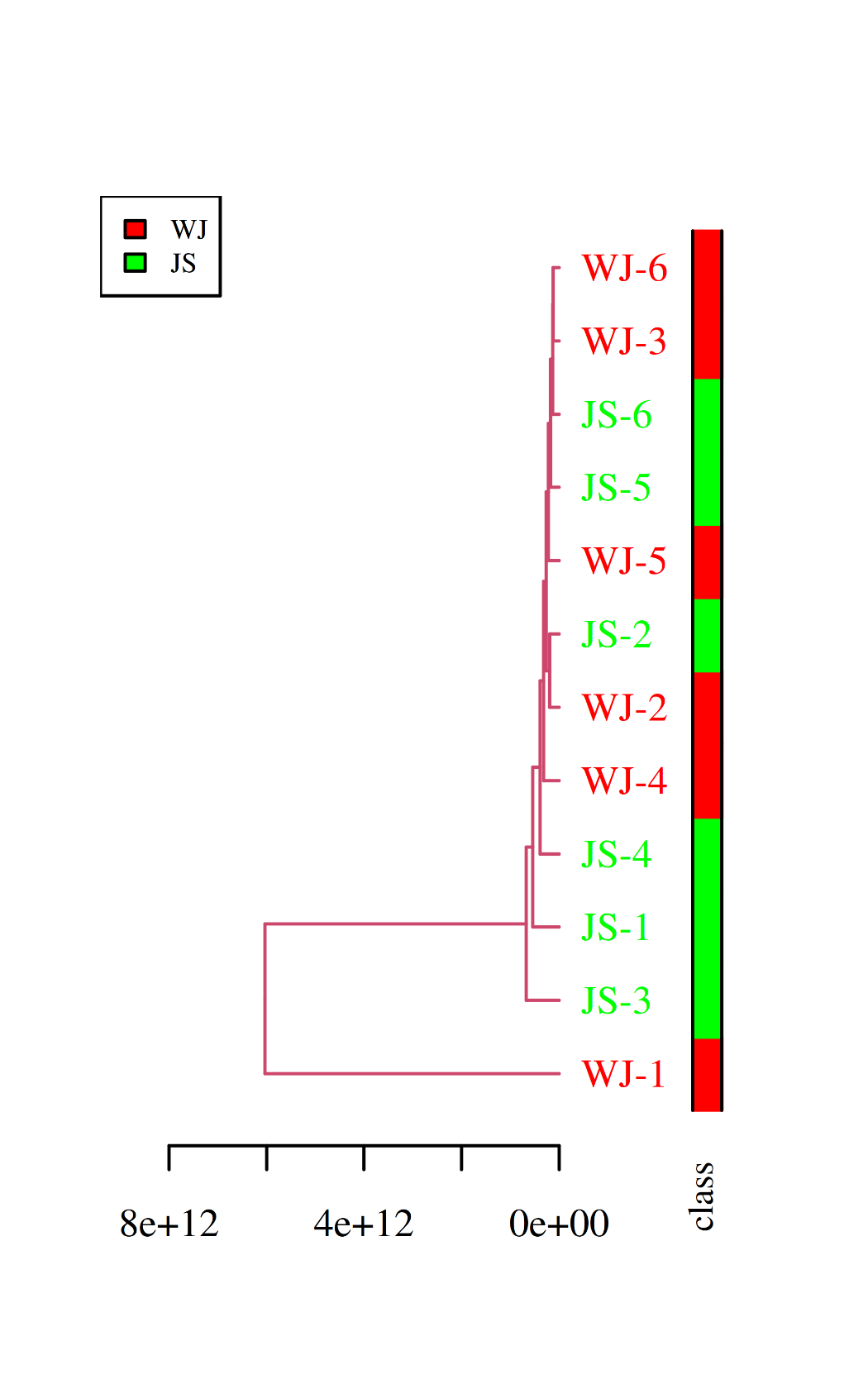


Figure S6 Dendrogram of the overall samples in negative ion mode

Supplement: S6 Fig — (DOCX) [file pone.0310786.s006.docx]

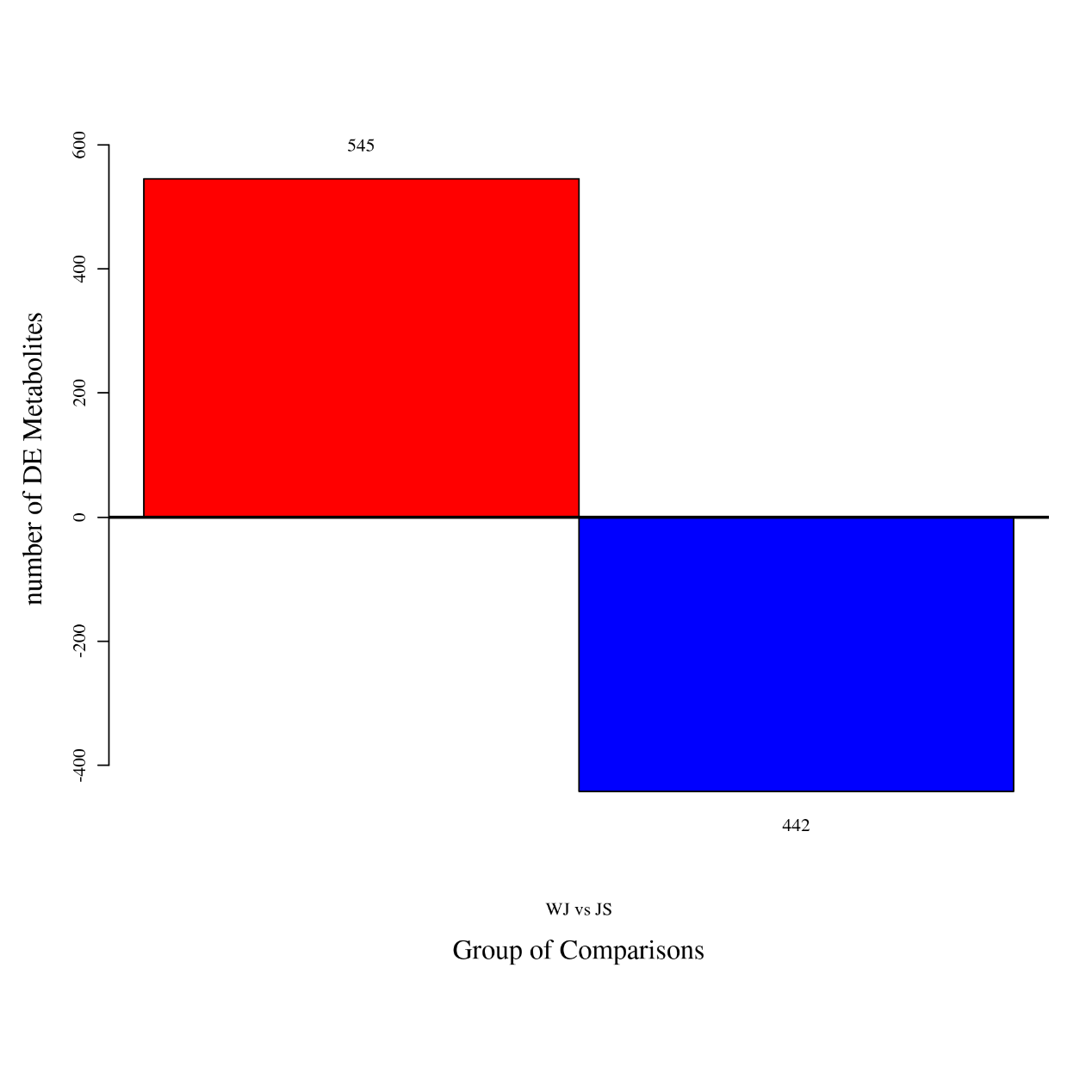


Figure S7 Total differential metabolites in positive ion mode

Supplement: S7 Fig — (DOCX) [file pone.0310786.s007.docx]

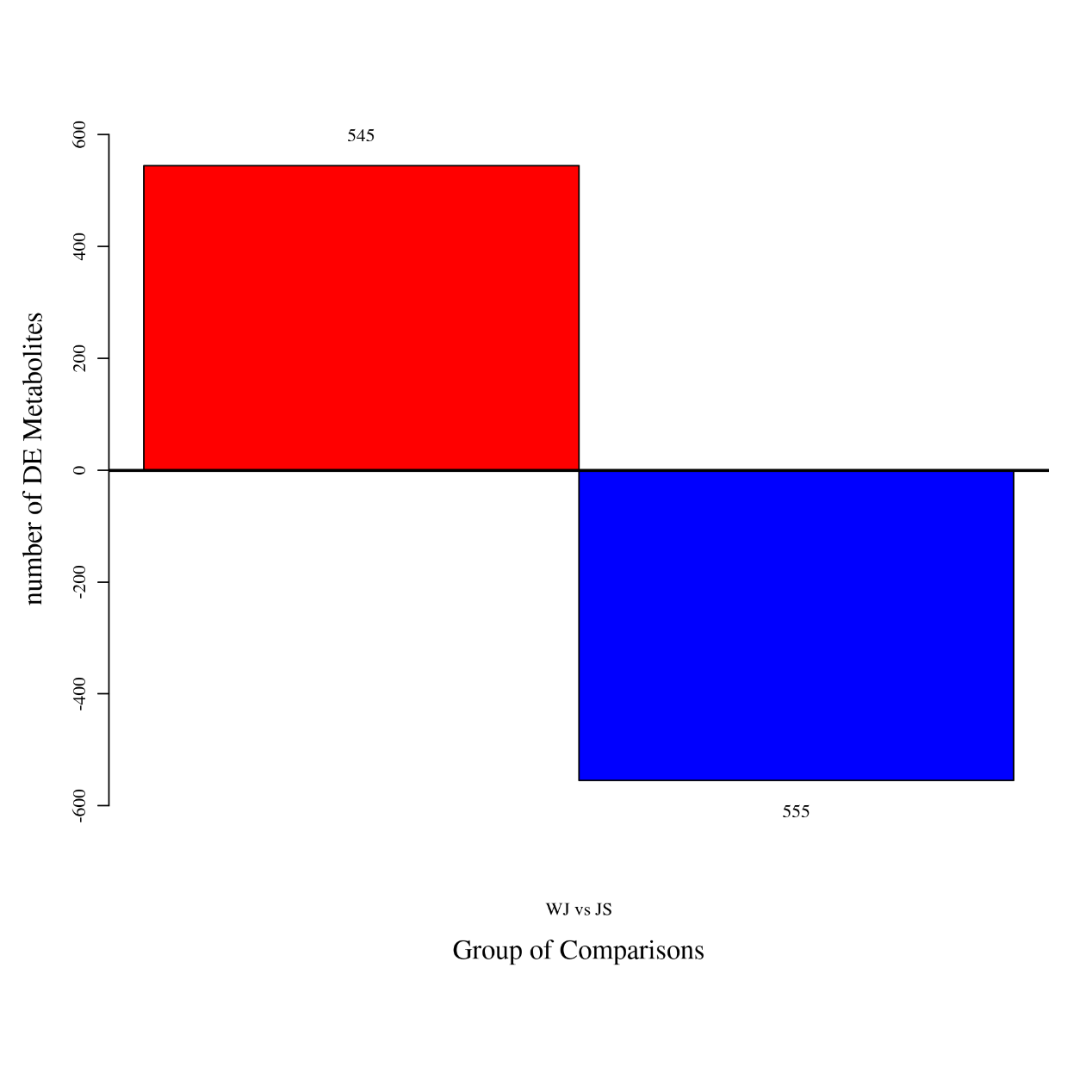


Figure S8 Total differential metabolites in negative ion mode

Supplement: S8 Fig — (DOCX) [file pone.0310786.s008.docx]

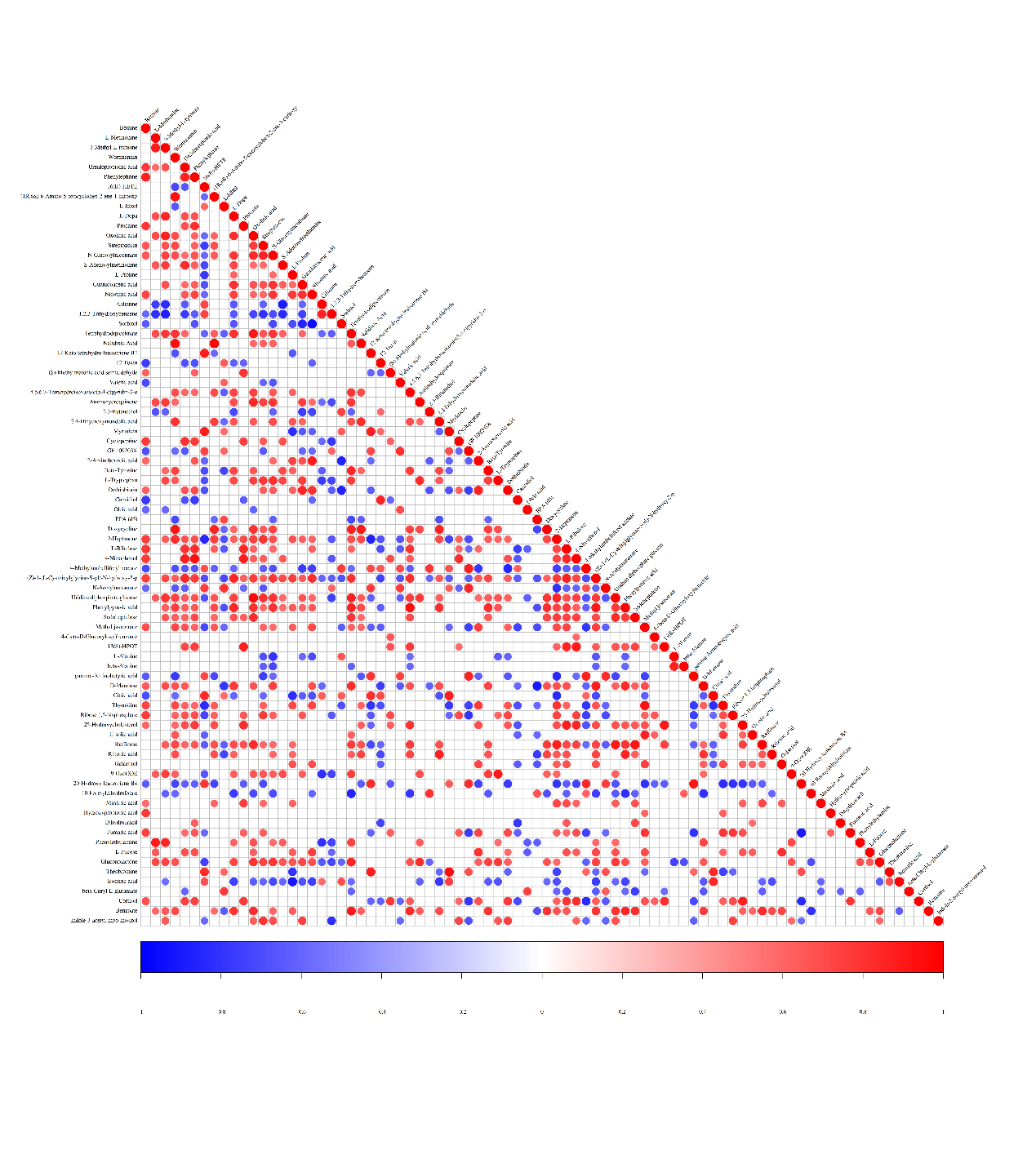


Figure S9 Correlation heatmap of differential metabolites of WJ and JS samples

Supplement: S9 Fig — (DOCX) [file pone.0310786.s009.docx]
