# Supplementary material for: Host plants selection of Centranthera grandiflora Benth. and nontargeted metabolomics analysis of its parasitic and non-parasitic samples
Source: PLoS One. 2025 Feb 5;20(2):e0310786. doi: 10.1371/journal.pone.0310786 (PMC11798490; doi:10.1371/journal.pone.0310786)
Supplement: Attachment — Submitted filename: Response to Reviewers.docx [file pone.0310786.s011.docx]

**Dear editors and reviewers,**

On behalf of my co-authors, we thank you very much for giving us an opportunity to revise our manuscript entitled “Host plants selection of *Centranthera grandiflora* Benth. and nontargeted metabolomics analysis of its parasitic and non-parasitic samples”. We appreciate editors and reviewers for your positive and constructive comments and suggestions on our manuscript. We have studied the comments carefully and have made corrections which we hope meet with approval. Revised portion are marked in red using the track changes mode in MS Word and the responds to the reviewer’s comments in the paper are as follows:

**Response to editors:**

**1. Please ensure that your manuscript meets PLOS ONE's style requirements, including those for file naming. The PLOS ONE style templates can be found at**

**https://journals.plos.org/plosone/s/file?id=wjVg/PLOSOne_formatting_sample_main_body.pdf and**

**https://journals.plos.org/plosone/s/file?id=ba62/PLOSOne_formatting_sample_title_authors_affiliations.pdf**

**Response:** Special thanks for your kind suggestion. We have revised our manuscript according to the two style templates.

**2. Please amend the title to remove the date: "Title. Host plants selection of Centranthera grandiflora Benth. and nontargeted metabolomics analysis of its parasitic and non-parasitic samples" should be "Host plants selection of Centranthera grandiflora Benth. and nontargeted metabolomics analysis of its parasitic and non-parasitic samples"**

**Response:** Thanks for your suggestion. The title has been removed in the manuscript.

**3. Please note that funding information should not appear in any section or other areas of your manuscript. We will only publish funding information present in the Funding Statement section of the online submission form. Please remove any funding-related text from the manuscript.**

**Response:** Special thanks for your kind suggestion. The funding information in the manuscript has been removed.

**4. Thank you for stating the following financial disclosure: "the Regional Grant of National Natural Science Foundation of China (Grant number 32360098); Youth Grant of National Natural Science Foundation of China (Grant number 32001684); Key Project of Yunnan Local University Joint Fund (Grant number 202201AN070036) and General Program of Yunnan Fundamental Research Projects (Grant number 202201AT070020)." Please state what role the funders took in the study. If the funders had no role, please state: "The funders had no role in study design, data collection and analysis, decision to publish, or preparation of the manuscript." If this statement is not correct you must amend it as needed. Please include this amended Role of Funder statement in your cover letter; we will change the online submission form on your behalf.**

**Response:** Special thanks for your kind suggestion. The amended Role of Funder statement was included and marked red in our cover letter.

**5. We note that your Data Availability Statement is currently as follows: All relevant data are within the manuscript and its Supporting Information files. Please confirm at this time whether or not your submission contains all raw data required to replicate the results of your study. Authors must share the “minimal data set” for their submission. PLOS defines the minimal data set to consist of the data required to replicate all study findings reported in the article, as well as related metadata and methods (https://journals.plos.org/plosone/s/data-availability#loc-minimal-data-set-definition).**

**For example, authors should submit the following data:**

**- The values behind the means, standard deviations and other measures reported;**

**- The values used to build graphs;**

**- The points extracted from images for analysis.**

**Authors do not need to submit their entire data set if only a portion of the data was used in the reported study.**

**If your submission does not contain these data, please either upload them as Supporting Information files or deposit them to a stable, public repository and provide us with the relevant URLs, DOIs, or accession numbers. For a list of recommended repositories, please see https://journals.plos.org/plosone/s/recommended-repositories.**

**If there are ethical or legal restrictions on sharing a de-identified data set, please explain them in detail (e.g., data contain potentially sensitive information, data are owned by a third-party organization, etc.) and who has imposed them (e.g., an ethics committee). Please also provide contact information for a data access committee, ethics committee, or other institutional body to which data requests may be sent. If data are owned by a third party, please indicate how others may request data access.**

**Response:** Special thanks for your kind suggestion. We confirmed that our submission contains all raw data required to replicate the results of our study.

**6. When completing the data availability statement of the submission form, you indicated that you will make your data available on acceptance. We strongly recommend all authors decide on a data sharing plan before acceptance, as the process can be lengthy and hold up publication timelines. Please note that, though access restrictions are acceptable now, your entire data will need to be made freely accessible if your manuscript is accepted for publication. This policy applies to all data except where public deposition would breach compliance with the protocol approved by your research ethics board. If you are unable to adhere to our open data policy, please kindly revise your statement to explain your reasoning and we will seek the editor's input on an exemption. Please be assured that, once you have provided your new statement, the assessment of your exemption will not hold up the peer review process.**

**Response:** Special thanks for your kind suggestion. All the authors agreed on the sharing plan.

**Response to Reviewer #1:**

**1. I find this study interesting. However, I cannot fully assess the overall content of the manuscript due to the complexity of the results and discussion sections. The authors combine the results and discussion, followed by a separate discussion, which makes it difficult to follow. I suggest improving the presentation by clearly separating the results and discussion sections. The results section should exclusively focus on presenting the findings. Any specific methods or approaches used should be detailed in the methods section. Discussions on the significance of the results should be confined to the discussion section, connecting the overall findings. Currently, I do not find the manuscript to be well-organized or clearly presented. I recommend that the authors enhance the overall quality of the writing.**

**Response:** Special thanks for your good suggestion. The manuscript was revised by a native English speaker in both writing quality and organizational structure to meet the publication criteria. And to make the article clear, we added two pictures (Figure 7 and Figure 8) and adjusted Table 3 for easy understanding. Figure 7 and Figure 8 were listed below.

Figure 7 Bubble plots of the metabolic pathway analysis. The main metabolic pathways were labeled in the figure according to the pathway impact and -log(P) values

Figure 8 the pathways for the main efficacy components of *C. grandiflora* and their possible competitive pathways (purple)

**If you are considering a plant parasitic study, analyzing the chloroplast genome would be highly beneficial. I suggest comparing the chloroplast genomes of parasitic and non-parasitic plants within the same family. The chloroplast genome for the species in question is already available. It is known that due to the parasitic nature, the chloroplast genome is under reduced selective pressure, leading to degradation.**

**Hopefully, you will add this in all parts of manuscript.**

**Response:** Special thanks for your good suggestion. According to your good suggestion, we have reviewed a large number of literatures on chloroplast genome research, and have gained a preliminary understanding of chloroplast genome related research. Analyzing the chloroplast genomes of parasitic and non-parasitic plants in the same family is of great significance to the phylogenetic analysis and species identification of *Scrophulariaceae* plants, which will be very beneficial to our research. However, we have not been able to find the sequence and annotation information of chloroplast genome of plants similar to *Centranthera grandiflora* Benth in NCBI database after searching in many different ways. In addition, due to the long cultivation cycle of *Centranthera grandiflora* Benth, it is difficult for us to complete the sequencing and assembling of the chloroplast genome of the plant in the short term. We plan to complete the sequencing and assembling of the chloroplast genomes of the plants in accordance with your suggestion, so as to provide a theoretical basis for species identification and resource development and utilization of *Scrophulariaceae* plants.

**Response to Reviewer #2:**

**This manuscript assesses the host compatibility for *C. grandiflora* and contrasts the metabolic profiles of parasitized versus non-parasitized *C. grandiflora* plants. Collectively, the findings contribute to a deeper understanding of *C. grandiflora*'s growth dynamics, which is instrumental for guiding its cultivation and application strategies moving forward. Nonetheless, the manuscript's current presentation requires** **refinement in both writing quality and organizational structure to meet the publication criteria.**

**Response:** Special thanks for your good suggestion. The manuscript was revised in both writing quality and organizational structure by a native English speaker to meet the publication criteria.

**1. Line Numbers: Please include line numbers in the manuscript to enhance the navigability and efficiency of the review process.**

**Response:** Special thanks for your good suggestion. Line numbers were included in the manuscript.

**2. Paragraph Clarification: On page 3, the secondary paragraph contains a passage that requires rephrasing for clarity and correction of typographical errors: "...2-year-old seedlings with soil ball perennial roots [4]. the leaves of ...".**

**Response:** Special thanks for your good suggestion. The paragraph has been rephrased, and it was changed into “in the case of seedlings, seedling in culture, or 2-year-old rooted seedling with soil ball [4]. The leaves of ...” and was marked red.

**3. Table 1 Correction: In Table 1, there is a typographical error in the column name; "Name of the possible host palnts" should be corrected to "Name of the possible host plants".**

**Response:** Thanks for your kind correction. We are very sorry for our carelessness.

**4. Figure and Table Legends: The legends for figures and tables should be expanded with more detailed descriptions to improve clarity and understanding.**

**Response:** Special thanks for your good suggestion. Some of the legends for figures and tables have been expanded and marked red in the article. For example, Table 3 “Identification information of differential metabolites between JS and WJ samples” was changed to “Identification information of the 82 differential metabolites between JS and WJ samples” and in the table, the number of up-regulated compounds of JS and WJ samples were labeled in detail.

**5. Consistency in Formatting: Ensure consistency in the use of spaces around the degree symbol for temperature indications, such as "-20 ℃" or "25 ℃", throughout the manuscript.**

**Response:** Special thanks for your kind suggestion. The use of spaces around the degree symbol for temperature indications were changed for consistency throughout the manuscript as “xx ℃”

**6. Figure 2 Enhancement: The inclusion of a scale bar in Figure 2 may aid reviewers in better grasping the spatial context presented in the image.**

**Response:** Special thanks for your good suggestion. The scale bar was included in Figure 2 as follows. Thanks again for your kind advice.

Figure 2 Haustoria connections between *C. grandiflora* and the host (red: roots of *C. grandiflora*, black: roots of the host)

**7. Clarification Needed: On page 12, in the subhead "Differential analysis of parasitic (JS) and non-parasitic (WJ) C. grandiflora", specify the aspects in which the differences are being analyzed. And also check through the manuscript.**

**Response:** Special thanks for your kind suggestion. We were deeply sorry that the subhead was not match with the following contents. We’ve changed the subhead into “OPLS-DA analysis of metabolites in JS and WS samples” and marked red in the article.

**8. Figure Title Formatting: The use of parentheses in figure titles, as seen in Figures 4 and 5, is not recommended.**

**Response:** Special thanks for your kind suggestion. The title of A and B in the parentheses has been changed to left and right for distinguishment.

**9. Capitalization Consistency: Inconsistencies in capitalization have been noted in various sections, such as "****Organic oxygen" and "Lipids" on page 14, which should be corrected for a professional presentation.**

**Response:** Special thanks for your kind suggestion. The inconsistencies in capitalization has been revised throughout the whole article and marked red.

**10. Table Presentation: The design of most tables should be refined to achieve a higher standard of formality and visual appeal.**

**Response:** Special thanks for your kind suggestion. All the tables have been refined to make the manuscript more clear. Besides, two figures (Figure 7 and Figure 8) were added for easy understanding of Table 4. Figure 7 and Figure 8 were listed below.

Figure 7 Bubble plots of the metabolic pathway analysis. The main metabolic pathways were labeled in the figure according to the pathway impact and -log(P) values

Figure 8 the pathways for the main efficacy components of *C. grandiflora* and their possible competitive pathways (purple)
