# Supplementary material for: Host plants selection of Centranthera grandiflora Benth. and nontargeted metabolomics analysis of its parasitic and non-parasitic samples
Source: PLoS One. 2025 Feb 5;20(2):e0310786. doi: 10.1371/journal.pone.0310786 (PMC11798490; doi:10.1371/journal.pone.0310786)
Supplement: Attachment — Submitted filename: Response to Reviewers0902.docx [file pone.0310786.s012.docx]

**Dear editors and reviewers,**

On behalf of my co-authors, we thank you very much for giving us an opportunity to revise our manuscript entitled “Host plants selection of *Centranthera grandiflora* Benth. and nontargeted metabolomics analysis of its parasitic and non-parasitic samples”.

This article represents a comprehensive study on the cultivation and metabolic aspects of the rare ethnic medicinal plant, *Centranthera grandiflora* Benth., in Yunnan Province. To the best of our knowledge as the authors, the content herein is deemed exhaustive and well-rounded. This plant is on the verge of extinction in China and the world, due to the fact that no artificial cultivation method has been found for a long time, and it is also classified as a rare plant in China. Our research team has spent nearly a decade uncovering the hemiparasitic nature of the *Centranthera grandiflora* Benth., thereby enabling its successful artificial cultivation. Currently, there is limited research on the growth and metabolism of hemiparasitic plants, making this article a highly valuable piece for publication and sharing. The article could provide some references for exploring the growth and metabolism mechanisms of hemiparasitic plants. However, although our team has achieved the artificial cultivation of *Centranthera grandiflora* Benth., the cultivation methods still require further refinement to facilitate large-scale planting and subsequently enable more scientific researches, which is a long-term work.

We have diligently addressed each and every comment from the reviewers, and the subsequent responses from the new set of reviewers have not surfaced any specific concerns or issues with the manuscript. Therefore, we humbly request the editor's final scrutiny to review our revised submission and kindly inform us of any necessary revisions or adjustments that may be required. Your invaluable insights and guidance would be greatly appreciated. Thank you for your attention and efforts in this regard.

Sincerely yours,

Liu He
